# Mitochondrial Antiviral Signaling Protein Activation by Retinoic Acid-Inducible Gene I Agonist Triggers Potent Antiviral Defense in Umbilical Cord Mesenchymal Stromal Cells Without Compromising Mitochondrial Function

**DOI:** 10.3390/ijms26104686

**Published:** 2025-05-14

**Authors:** Sebastián Castillo-Galán, Felipe Grünenwald, Yessia Hidalgo, J César Cárdenas, Maria Ignacia Cadiz, Francisca Alcayaga-Miranda, Maroun Khoury, Jimena Cuenca

**Affiliations:** 1 Centro de Investigación e Innovación Biomédica (CIIB), Universidad de los Andes, Santiago 7550000, Chile; se.castillo.galan@gmail.com (S.C.-G.); ext_fgrunenwald@uandes.cl (F.G.); yhidalgo@uandes.cl (Y.H.); falcayaga@uandes.cl (F.A.-M.); mkhoury@uandes.cl (M.K.); 2 IMPACT, Center of Interventional Medicine for Precision and Advanced Cellular Therapy, Santiago 7550000, Chile; 3 Center for Integrative Biology, Faculty of Sciences, Universidad Mayor, Santiago 750000, Chile; julio.cardenas@umayor.cl; 4 Geroscience Center for Brain Health and Metabolism, Santiago 7750000, Chile; 5 Department of Chemistry and Biochemistry, University of California, Santa Barbara, CA 93101, USA; 6 Cells for Cells, Santiago 7550000, Chile; micadiz@c4c.cl; 7 Consorcio REGENERO, The Chilean Consortium for Regenerative Medicine, Santiago 8330024, Chile

**Keywords:** mesenchymal stromal cells, viral RNA, MAVS, RIG-I agonist, cellular and mitochondrial function

## Abstract

Mesenchymal stromal cells (MSCs) represent a promising therapeutic approach in viral infection management. However, their interaction with viruses remains poorly understood. MSCs can support antiviral immune responses and act as viral reservoirs, potentially compromising their therapeutic potential. Innate immune system recognition of viral pathogens involves pattern recognition receptors (PRRs), including RIG-I-like receptors (RLRs), which activate mitochondrial antiviral signaling protein (MAVS). MAVS triggers antiviral pathways like IRF3 and NF-κB, leading to interferon (IFN) production and pro-inflammatory responses. This study explores the antiviral response in umbilical cord-derived MSCs (UC-MSCs) through targeted stimulation with influenza A virus-derived 5′triphosphate-RNA (3p-hpRNA), a RIG-I agonist. By investigating MAVS activation, we provide mechanistic insights into the immune response at the molecular level. Our findings reveal that 3p-hpRNA stimulation triggers immune activation of the IRF3 and NF-κB pathways through MAVS. Subsequently, this leads to the induction of type I and III IFNs, IFN-stimulated genes (ISGs), and pro-inflammatory cytokines. Critically, this immune activation occurs without compromising mitochondrial integrity. UC-MSCs retain their capacity for mitochondrial transfer to recipient cells. These results highlight the adaptability of UC-MSCs, offering a nuanced understanding of immune responses balancing activation with metabolic integrity. Finally, our research provides mechanistic evidence for MSC-based interventions against viral infections.

## 1. Introduction

Mesenchymal stromal cells (MSCs) have emerged as a promising cell-based therapy due to their immunomodulatory and regenerative properties [1]. These cells have been widely evaluated in different preclinical settings and clinical applications [2,3,4,5], including virus-associated diseases such as COVID-19 [6,7]. However, the interplay between MSCs and viruses is like a double-edged sword. MSCs can support antiviral immune responses by promoting the proliferation and function of antiviral-specific effector cells and exerting antimicrobial activity [8]; conversely, they may act as viral reservoirs or be susceptible to infection [9,10,11], potentially compromising their therapeutic efficacy [12,13]. Although several preclinical studies on viral-infected models have shown the therapeutic effects of different tissue-derived MSCs [14,15], conflicting findings indicate that their role in viral infections remains underexplored and highlight the need to understand MSC–virus interactions better [7].

The innate immune system serves as the first line of defense against pathogens. Cells express pattern recognition receptors (PRRs) that detect pathogen-associated molecular patterns (PAMPs, e.g., viral nucleic acids), leading to the activation of intracellular signaling pathways and induction of the antiviral response [16,17]. Within the PRRs, the retinoic acid-inducible gene I (RIG-I)-like receptors (RLRs) are critical in detecting cytosolic viral RNA. This family comprises three members: RIG-I, MDA5 (melanoma differentiation-associated gene 5), and LGP2 (laboratory of genetics and physiology 2) [18]. Upon sensing viral RNA, RIG-I and MDA5 initiate downstream signaling by interacting with the mitochondrial antiviral signaling protein (MAVS), which forms prion-like aggregates that convert other MAVS on the mitochondrial outer membrane into prion-like aggregates [19]. This MAVS activation triggers an antiviral signaling cascade that induces interferon (IFN) regulatory factors (IRFs) and nuclear factor kappa B (NF-κB), leading to the transcription of IFN-stimulated genes (ISGs) and inflammatory cytokines [20,21,22,23,24].

MSCs recognize viral pathogens through PRRs and modulate antiviral defenses by upregulating ISGs and inflammatory cytokine production [25]. For instance, Yu et al. demonstrated that adipose-derived MSCs express functional PRRs, including RIG-I and TLR3, which recognize viral nucleic acids and induce antiviral immune responses [26]. Similarly, Yang et al. showed that activating RLR pathways in mouse BM-MSCs triggers IRF3 and NF-κB signaling, leading to cytokine production and immune regulation [27]. In addition, Wu et al. reported that MSCs exhibit constitutive expression of ISGs, providing them with an intrinsic antiviral resistance that distinguishes them from differentiated cells [28]. Despite these advances, critical gaps remain in understanding MAVS activation in UC-MSCs, particularly its impact on cell and mitochondrial function and antiviral signaling.

Mitochondria are essential to MAVS-dependent antiviral signaling, requiring a precise balance between activation and homeostasis to ensure effective immune responses. A recent study proposes that MSCs engineered to overexpress MAVS, particularly in combination with viral S protein expression, could enhance the innate immune response and IFN production in a targeted manner [29]. Further research is needed to define strategies for modulating MAVS activation in MSCs and assess its implications for advanced cell therapies. Additionally, MSC-mediated mitochondrial transfer to host cells has been shown to enhance cellular bioenergetics and promote tissue repair in several preclinical models [30]. Nevertheless, how this process is impacted in a viral context remains unclear.

This study explores how MAVS activation in UC-MSCs, triggered by influenza A virus (IAV)-derived 5′triphosphate-RNA, a potent RIG-I agonist, influences antiviral signaling and mitochondrial function. Our goal was to determine whether MAVS activation enhances the antiviral capacity of UC-MSCs while maintaining their metabolic stability. Understanding these molecular mechanisms could shed light on how UC-MSCs contribute to innate antiviral immunity, particularly in the context of RNA respiratory viruses such as IAV, respiratory syncytial virus (RSV), and certain human coronaviruses (e.g., SARS-CoV-2). We pave the way for developing novel cell-based strategies that modulate inflammation and restore antiviral defense in severe respiratory viral infections—a clinical scenario where current treatments remain largely supportive and fail to address the underlying immune dysfunction.

## 2. Results

### 2.1. Influenza a Virus RNA (3p-hpRNA) Acts as a RIG-I Agonist, Inducing MAVS Expression and Activation in UC-MSCs

**Figure 1:**
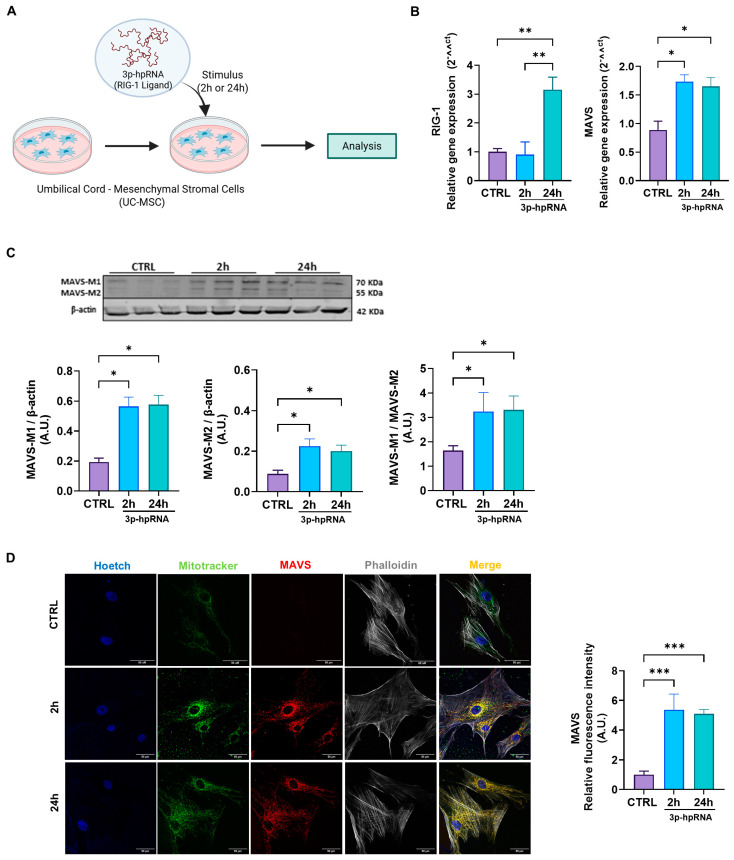
Stimulation with the 3p-hpRNA increased MAVS activation in UC-MSCs. (**A**) Experimental scheme. UC-MSCs were stimulated for 2 and 24 h with 250 ng/mL of 3p-hpRNA, a RIG-I agonist. Created with BioRender.com. (**B**) Relative gene expression (RT-qPCR) of RIG-I and MAVS. (**C**) Representative Western blot image and densitometry of MAVS-M1, M2 isoforms, and β-actin, respectively, and ratio of MAVS-M1/MAVS-M2, normalized to β-actin. (**D**) Representative confocal microscopy images (62X) of stimulated UC-MSCs and semi-quantification of fluorescence intensity of MAVS. Nucleus (blue), MitoTracker green (green), MAVS (red), and Phalloidin (white). Bars represent mean +/− SEM; three different UC-MSC donors; three independent experiments; * *p* ≤ 0.05; ** *p* ≤ 0.01, *** *p* ≤ 0.001. One-way ANOVA, Benjamini–Hochberg FDR. A.U.: arbitrary units; UC-MSC: umbilical cord-mesenchymal stromal cells; MAVS: mitochondrial antiviral signaling protein; RIG-I: retinoic acid-inducible gene-I.

MAVS plays a critical adaptor molecule in the innate immune response against viral RNA. To assess whether MAVS pathway activation influences the functional properties of UC-MSCs and their mitochondria, we first evaluated the pathway’s activation at distinct time points (Figure 1A, experimental model). Specifically, we examined the effect of IAV-derived 5′triphosphate-RNA or 3p-hpRNA [31] on the gene expression of RIG-I, observing a significant increase after 24 h of stimulation versus the control (Figure 1B). Since 3p-hpRNA acts as a selective agonist of RIG-I [31] and does not activate other double-stranded RNA sensors, such as TLR3 or MDA5, it provides a precise experimental model for delineating MAVS pathway activation. Both gene and protein expression levels of MAVS were consistently elevated by approximately twofold at 2 and 24 h following 3p-hpRNA stimulation (Figure 1B,C). Furthermore, the ratio between MAVS-M1 and MAVS-M2 isoforms increased by three times after 2 and 24 h of stimulation (1.64 A.U. ± 0.19 vs. 3.23 A.U. ± 0.78 vs. 3.31 A.U. ± 0.57 for CTRL, 2 and 24 h, respectively) (Figure 1C). These findings suggest that the MAVS increase is closely linked to the activation of RIG-I in this context.

MAVS is predominantly localized within the mitochondrial network, a critical feature for its role in antiviral signaling. To investigate its localization, we performed confocal microscopy on UC-MSCs stained with MitoTracker Green (to visualize mitochondria) and an anti-MAVS antibody (to detect MAVS localization). The merged images revealed a clear colocalization of MAVS with mitochondria, as indicated by yellow signals in the overlapping regions, substantially elevated after stimulation with 3p-hpRNA at 2 and 24 h (1.00 ± 0.24 vs. 5.37 ± 1.06 vs. 5.09 ± 0.29 for CTRL, 2 and 24 h, respectively) (Figure 1D), suggesting that MAVS is effectively positioned within the mitochondrial network to mediate antiviral responses.

### 2.2. p-hpRNA Activates Both IRF3 and NF-κB Signaling in UC-MSCs

Next, to investigate the antiviral response associated with MAVS activation, we assessed the signaling pathways involving IRF3 and NF-κB, which are crucial to producing IFNs and pro-inflammatory cytokines.

**Figure 2:**
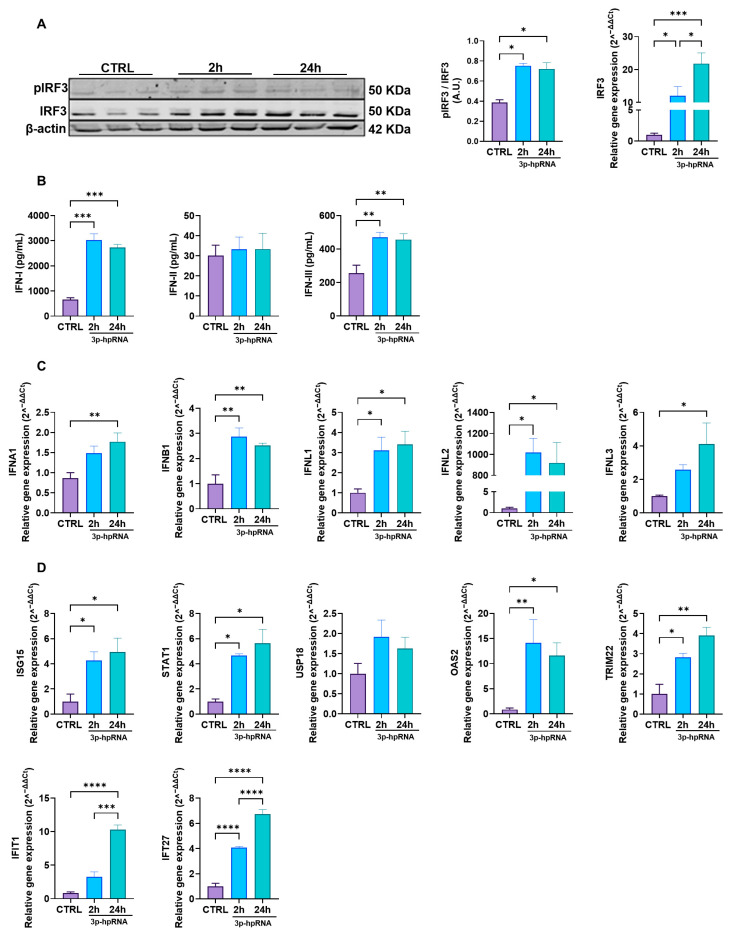
3p-hpRNA activates the IRF3 pathway in UC-MSCs. UC-MSCs were stimulated for 2 and 24 h with 250 ng/mL of 3p-hpRNA, a RIG-I agonist. (**A**) Representative image of Western blot to detect phospho (p-) and total IRF3 at 2 and 24 h post-stimulus. Densitometry of p-IRF3/IRF3 expression, normalized to β-actin, and relative gene expression of IRF3 normalized to β-actin. (**B**) Production (ELISA) of type I IFNs (IFN-α and β), type II IFN (IFN-γ), and type III IFNs (IFN λ1, λ2, and λ3) at different time points post-stimulation. (**C**) Relative gene expression of IFNs: type 1 (IFNA1 and IFNB1), type II IFN (IFNL1 and IFNL2), and type III IFNs (IFNL3) at different time points post-stimulation and normalized to β-actin. (**D**) Relative gene expression of IFN-stimulated genes (ISGs): ISG15, STAT1, USP18, OAS2, TRIM22, IFIT1, and IFI27 at different time points. Gene expression was normalized to β-actin as housekeeping. Bars represent mean +/−SEM; three different UC-MSC donors; three independent experiments; * *p* ≤ 0.05; ** *p* ≤ 0.01; *** *p* ≤ 0.001; **** *p* ≤ 0.0001; One-way ANOVA, Benjamini–Hochberg FDR. A.U.: arbitrary units. IRF3: interferon regulatory factor 3; IFN: interferon; ISG15: interferon-stimulated gene 15; STAT1: signal transducer and activator of transcription 1; USP18: ubiquitin-specific peptidase 18; OAS2: 2′-5′ oligoadenylate synthetases; TRIM22: tripartite motif containing 22; IFIT1: interferon-induced protein with tetratricopeptide repeats 1; IFI27: interferon alpha-inducible protein 27.

Upon stimulation with 3p-hpRNA, MAVS activates different kinases, ultimately resulting in the phosphorylation of IRF3 and transcriptional induction of type I and III IFNs and ISGs. As shown in the Western blotting in Figure 2A, IRF3 (pIRF3) phosphorylation increased almost twofold in UC-MSCs stimulated for 2 and 24 h compared to the unstimulated control (0.39 ± 0.02 vs. 0.75 ± 0.02 vs. 0.72 ± 0.06, for CTRL, 2 and 24 h, respectively). Correspondingly, IRF3 gene expression levels were also elevated at both time points compared with baseline levels (Figure 2A). ELISA analysis showed a significant increase in IFN-I and -III production at 2 and 24-h post-stimulation, whereas IFN-II (IFN-γ) levels remained unchanged (Figure 2B). Accordingly, the gene expression of type I and III IFNs, where UC-MSCs were stimulated with 3p-hpRNA, demonstrated a marked increase in the relative levels of IFN-α and β (type I) and IFNs-λ1, 2, and 3 (type III) (Figure 2C), consistent with their role in antiviral defense.

Moreover, the expression of ISGs revealed a significant upregulation compared to the control of interferon-stimulated gene 15 (ISG15), signal transducer and activator of transcription 1 (STAT1), 2′-5′-oligoadenylate synthetase 2 (OAS2), tripartite motif containing 22 (TRIM22), IFN-induced protein with tetratricopeptide repeats 1 (IFIT1), and IFN alpha inducible protein 27 (IFI27), following stimulation with 3p-hpRNA (Figure 2D). These ISGs play critical roles in viral recognition through PRRs (IFIT1) [32], suppression of viral replication by promoting viral RNA degradation (OAS2) [33], inhibition of viral protein translation (ISG15, IFIT1) [34], and interference with viral particle assembly (TRIM22) [35]. Furthermore, they contribute to regulating the innate immune response by activating key transcription factors (STAT1) [36], modulating inflammatory pathways (ISG15, TRIM22), and inducing apoptosis in infected cells (IFI27) [37], thereby enhancing antiviral defense mechanisms.

**Figure 3:**
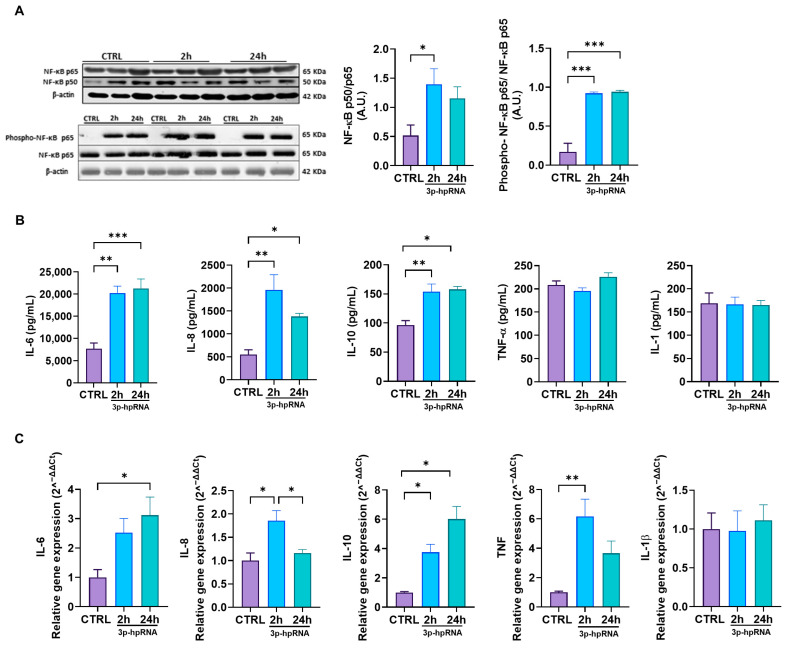
3p-hpRNA stimulation activated the NF-κB pathway in UC-MSCs. UC-MSCs were stimulated for 2 and 24 h with 250 ng/mL of 3p-hpRNA, a RIG-I agonist. (**A**) Representative image of Western blot and densitometry ratio of NF-κB (p50/p65) and NF-κB activation (phospho-p65/p65) with β-actin as a normalizer. (**B**) Production (ELISA) and (**C**) relative gene expression by RT-qPCR of cytokines induced by NF-κB (IL-6, IL-8, IL-10, TNF-α, and IL-1). Gene expression was normalized to β-actin for housekeeping. Bars represent mean +/− SEM; three different UC-MSC donors; three independent experiments; * *p* ≤ 0.05; ** *p* ≤ 0.01; *** *p* ≤ 0.001. One-way ANOVA, Benjamini–Hochberg FDR. A.U.: arbitrary units; RIG-I: retinoic acid-inducible gene-I; NF-κB: nuclear factor kappa-light-chain-enhancer of activated B cells; IL: interleukin; TNF-α: tumor necrosis factor alpha.

Complementary, we evaluated the activity of the NF-κB signaling pathway. The results showed that stimulation with 3p-hpRNA increases the NF-κB heterodimer p50/p65 compared to controls (Figure 3A). Furthermore, the Western blotting results also revealed increased phosphorylation of NF-κB p65 in the stimulated cells (Figure 3A), which could facilitate the nuclear translocation of the p50/p65 heterodimer, enhancing its transcriptional activation and prolonging the inflammatory response [38,39]. These results correlate with the augmented expression and release of inflammatory cytokines, determined by ELISA and qRT-PCR, which are bona fide NF-κB downstream targets such as IL-6, IL-8, IL-10, and TNF. At the same time, IL-1 remained unchanged (Figure 3B,C).

These results collectively demonstrate that MAVS activation with 3p-hpRNA in UC-MSCs leads to a robust antiviral response mediated by IRF3 and NF-κB, characterized by the induction of type I and III IFNs, various ISGs, and pro-inflammatory cytokines.

### 2.3. p-hpRNA Stimuli Did Not Alter the Cell or Mitochondrial Functions in UC-MSCs

This study aimed to determine whether 3p-hpRNA stimulation would affect cellular and mitochondrial functions. Such evaluation is particularly relevant given that mitochondrial health is closely linked to overall cell viability and function. For instance, research has shown that a compromised mitochondrial function can lead to increased oxidative stress and apoptosis, ultimately affecting the therapeutic potential of stem cells.

**Figure 4:**
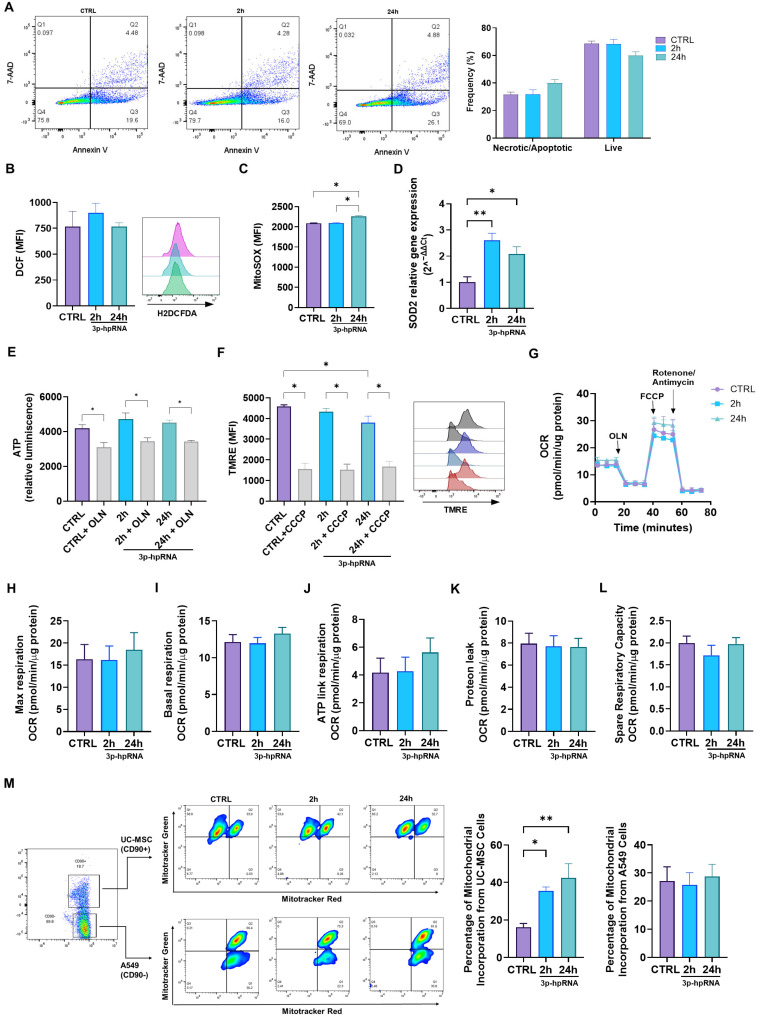
Effect of the 3p-hpRNA stimulation on the cellular and mitochondrial function in UC-MSCs. UC-MSCs were stimulated with 250 ng/mL of 3p-hpRNA, a RIG-I agonist (2 and 24 h). CTRL refers to the unstimulated cells. (**A**) Necrosis/apoptosis and viability were assessed by flow cytometry using annexin 5 and 7AAD staining; (**B**) reactive oxygen species (ROS) production was evaluated with DCF probe (H2DCFDA) and (**C**) MitoSOX by flow cytometry, and (**D**) relative gene expression of SOD2 by RT-qPCR. (**E**) ATP production was determined using the commercial kit, CellTiter-Glo^®^ Luminescent Cell Viability Assay. As a control, it was blocked with oligomycin (OLN, 1 µg/µL). Results were normalized to μg of total protein in each well. (**F**) The mitochondrial membrane potential (MMP) was evaluated through flow cytometry using the TMRE probe (Tetramethylrhodamine, Ethyl Ester, Perchlorate). Representative flow cytometry histogram (right). As a control, carbonyl cyanide m-chlorophenylhydrazone (CCCP) was used (10 µM for 10 min). (**G**) Seahorse assay results of OCR for UC-MSCs at 2 and 24 h post-stimulation. Results were normalized to µg of total protein in each well. (**H**) Maximal (Max) respiration, (**I**) basal OCR, (**J**) ATP-linked OCR, (**K**) protein leak, and (**L**) spare respiratory capacity of UC-MSCs at 2 and 24 h after stimulation. (**M**) Mitochondrial transfer between lung epithelial cells (A549) and UC-MSCs at 2 and 24 h after stimulation. Bars represent mean +/− SEM; three different UC-MSC donors; three independent experiments; * *p* ≤ 0.05, ** *p* ≤ 0.01. One-way ANOVA, Benjamini–Hochberg FDR. 7AAD: 7-aminoactinomycin D; SOD2: superoxide dismutase 2; OCR: oxygen consumption rate.

Our results revealed no differences in the levels of necrotic/apoptotic cells and live cells 24 h post-stimulation with 3p-hpRNA (Figure 4A). The oxidative stress levels (ROS) determined by the fluorescent sensor based on dichlorofluorescein (DCF) remained unchanged compared to the control groups (Figure 4B). Nevertheless, mitochondrial ROS (mtROS) levels (Figure 4C) and the expression of superoxide dismutase 2 (SOD2), a key mitochondrial enzyme involved in ROS detoxification, increased at 2 and 24 h after stimulation (Figure 4D).

Mitochondrial function was assessed by measuring mitochondrial membrane potential (ΔΨm), ATP levels, and cellular respiration. A slight decrease in ΔΨm was observed only at 24 h post-stimulation with 3p-hpRNA, while ATP levels remained stable across all groups, indicating preserved mitochondrial activity (Figure 4E,F). To further evaluate mitochondrial bioenergetics in UC-MSCs, the oxygen consumption rate (OCR) was analyzed using the Seahorse XF HS Mini Analyzer. Results showed that 3p-hpRNA stimulation had no impact on OCR, with basal respiration, ATP-linked respiration, maximal respiration, respiratory capacity, and proton leak remaining unchanged compared to controls (Figure 4G–L).

Finally, a co-culture system of UC-MSCs and lung epithelial cells (A549) demonstrated that mitochondrial transfer capacity is not compromised even after 3p-hpRNA stimulation (Figure 4M). As shown in Figure 4M, mitochondria derived from 3p-hpRNA-stimulated UC-MSCs were acquired by A549 cells at a higher percentage compared to those from unstimulated UC-MSCs. In contrast, the uptake of A549-derived mitochondria by UC-MSCs remained unchanged, regardless of prior stimulation with 3p-hpRNA.

These results suggest that 3p-hpRNA stimuli in UC-MSCs do not compromise mitochondrial bioenergetics or oxidative stress levels and preserve their capacity to transfer mitochondria to a cell recipient.

### 2.4. Mitochondrial Dynamics Are Not Affected by 3p-hpRNA Exposure

**Figure 5:**
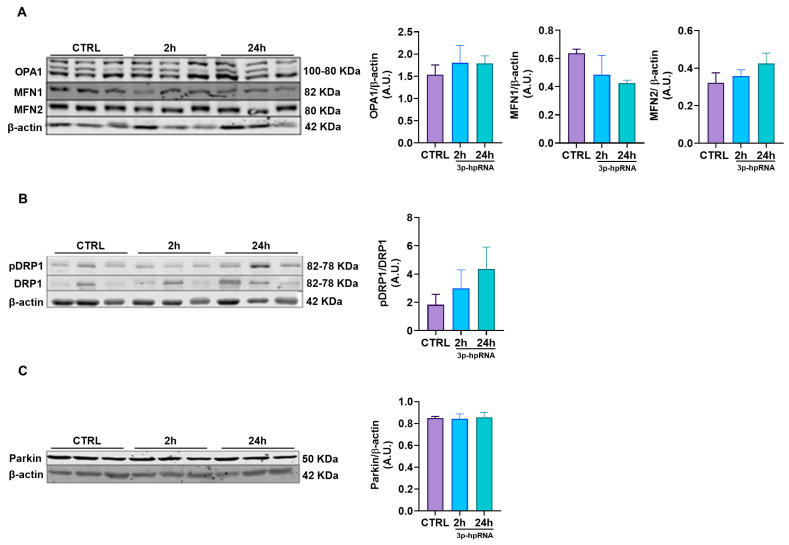
Effect of 3p-hpRNA on mitochondrial dynamics of UC-MSCs. UC-MSCs were stimulated with 250 ng/mL of 3p-hpRNA (2 and 24 h), and proteins involved in (**A**) fusion (OPA1, MFN1, MFN2), (**B**) fission (ratio pDRP1/DRP1), and (**C**) mitophagy (Parkin) were assessed. All proteins were normalized against β-actin. Bars represent mean +/− SEM, three different UC-MSC donors, three independent experiments. One-way ANOVA, Benjamini–Hochberg FDR. A.U.: arbitrary units; OPA1: optic atrophy type 1; MFN1: mitofusin 1; MFN2: mitofusin 2; DRP1: dynamin-related protein 1; pDRP1: phospho-dynamin-related protein 1.

Possible changes in mitochondrial morphology due to 3p-hpRNA stimulation were assessed by evaluating mitochondrial dynamics in UC-MSCs. The expression levels of proteins associated with fusion (Optic Atrophy 1, OPA1; Mitofusin, MFN1 and 2), fission (Dynamin-Related Protein 1, DRP1; Phosphorylated DRP1 pDRP1), and mitophagy (E3 Ubiquitin-Protein Ligase Parkin, PARKIN) were analyzed. No significant differences were observed in fusion markers (Figure 5A), fission markers (Figure 5B), or mitophagy markers (Figure 5C) at 2 and 24 h post-stimulus compared to the control group.

### 2.5. Isolated Mitochondria Derived from 3p-hpRNA-Stimulated UC-MSCs Maintain Their Morphology, ATP Production, Membrane Potential, and Adoption Capacity

**Figure 6:**
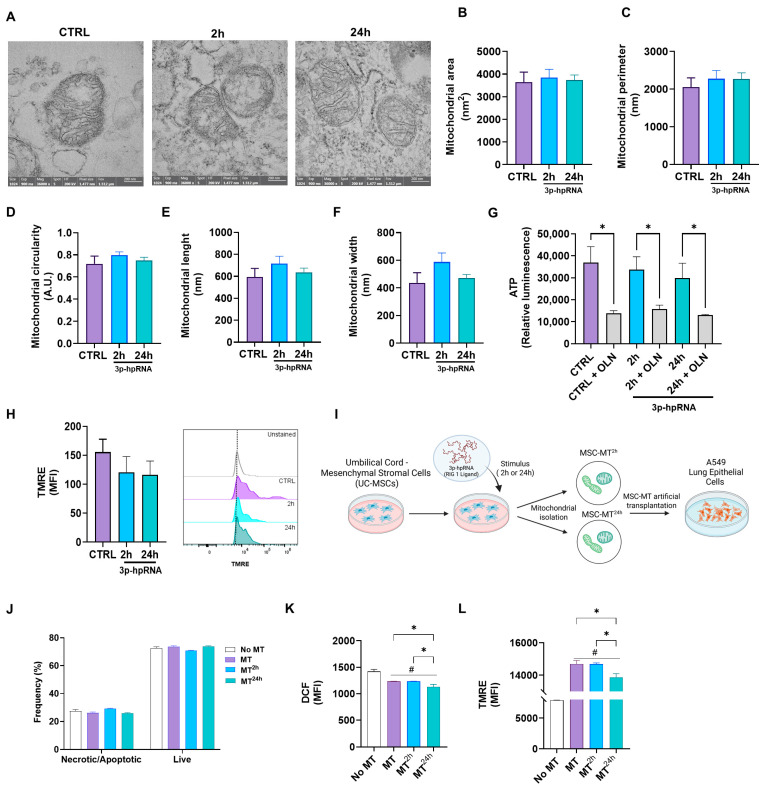
Effect of 3p-hpRNA agonist morphology and function in isolated mitochondria derived from UC-MSCs. UC-MSCs were stimulated for 2 and 24 h with 250 ng/mL of 3p-hpRNA, a RIG-1 I agonist. (**A**) Representative transmission electron microscopy (TEM) images of the conditions (naive mitochondria (MT) or control (CTRL) from MSCs and MSC-derived MT stimulated for 2 or 24 h). Determination of (**B**) area, (**C**) perimeter, (**D**) circularity, (**E**) length, and (**F**) width of mitochondria in TEM photographs, using the Image J analysis program. For these analyses, *n* = 44 were quantified, with 2 photographs per condition. (**G**) ATP production was determined using the commercial kit CellTiter-Glo^®^ Luminescent Cell Viability Assay. As a control, it was blocked with oligomycin (OLN, 1 µg/µL). Results were normalized to μg of total protein in each well. (**H**) Mitochondrial membrane potential was evaluated through flow cytometry using the TMRE probe (Tetramethylrhodamine, Ethyl Ester, Perchlorate). (**I**) Experimental scheme (created with Biorender.com): Mitochondrial enriched fractions isolated from naïve UC-MSCs (MT) or from UC-MSCs stimulated with 3p-hpRNA for 2 h (MT^2h^) or 24 h (MT^24h^), were transplanted into lung epithelial cells (A549), and (**J**) necrosis/apoptosis and viability were assessed by flow cytometry using Annexin V and 7AAD staining. (**K**) Reactive oxygen species (ROS) production was evaluated with DCF probe (H2DCFDA) by flow cytometry, and (**L**) mitochondrial membrane potential was evaluated through flow cytometry using the TMRE probe (Tetramethylrhodamine, Ethyl Ester, Perchlorate). Bars represent mean +/− SEM; * *p* ≤ 0.05; # *p* ≤ 0.05 vs. CTRL. One-way ANOVA, Benjamini–Hochberg FDR.

The analysis of preparations of isolated mitochondria provides a more focused and detailed understanding of mitochondrial function and bioenergetics, independent of cellular signaling cascades and other factors that regulate mitochondrial function. In this context, to evaluate the effects of 3p-hpRNA stimulation on mitochondria, mitochondrial morphology (Figure 6A–F), ATP production (Figure 6G), and ΔΨm (Figure 6H) were determined in mitochondrial-enriched fractions isolated from UC-MSCs at 2 and 24 h post-stimulation.

Transmission electron microscopy (TEM) is a direct method to assess the enrichment and morphological integrity of ex vivo mitochondrial preparations. In our samples, isolated mitochondria are easily identifiable as organelles with a double-membrane system and well-defined cristae (Figure 6A). The mitochondrial-enriched fraction obtained from UC-MSCs across different experimental groups exhibited no significant differences in area (Figure 6B), perimeter (Figure 6C), circularity (Figure 6D), length (Figure 6E), or width (Figure 6F) compared to controls. Likewise, ATP levels (Figure 6G) and ΔΨm (Figure 6H), determined by flow cytometry, remained comparable between 3p-hpRNA-stimulated groups and the control, indicating preserved mitochondrial integrity and bioenergetic function. On the other hand, to determine whether mitochondria derived from UC-MSCs stimulated with 3p-hprna exhibit any adverse effect on other host cells, mitochondria-enriched fractions were artificially transplanted (Mitoception) into lung epithelial cells (A549). Cell viability, oxidative stress levels, and ΔΨm were determined in these cells (Figure 6I–L).

A549 cells transplanted with mitochondria-enriched fractions derived from control UC-MSCs (MT) and UC-MSCs stimulated for 2 (MT^2h^) and 24 h (MT^24h^) with 3p-hpRNA showed similar percentages of necrosis/apoptosis and live cells in relation to the control (Figure 6J). Compared to controls without exogenous mitochondria, A549 cells transplanted with mitochondria-enriched fractions exhibited lower levels of oxidative stress (Figure 6K) and a higher membrane potential (Figure 6L).

## 3. Discussion

Mesenchymal stromal cells (MSCs) interact with their microenvironment and modulate immune responses through distinct mechanisms [8,40]. Although the immunomodulatory and antiviral properties of MSCs have been explored [7], few studies have examined how RIG-I and MAVS activation affect mitochondrial function and gene expression profiles in UC-MSCs [41,42].

This study demonstrated that stimulating UC-MSCs with 3p-hpRNA, a specific RIG-I agonist derived from the influenza A virus (H1N1) sequence [31], induces MAVS activation. This activation subsequently triggers the NF-κB and IRF3 pathways, upregulating ISGs and inflammatory cytokines. Despite this robust antiviral response, cellular and mitochondrial functions of UC-MSCs remained stable compared to unstimulated controls. This resilience suggests that UC-MSCs can generate an effective antiviral response without significantly compromising their viability, a crucial feature for their therapeutic potential.

Our results showed that 3p-hpRNA stimulation increased MAVS isoforms, with MAVS-M1 being the predominant active form. It is well-established that MAVS-M1 induces antiviral signaling, whereas MAVS-M2 acts as a negative regulator [43]. The increased gene expression of MAVS and RIG-I at 24 h post-stimulation aligns with findings in other cell types, including epithelial and dendritic cells, where peak expression occurs within 24–48 h after RNA-based agonist stimulation [44]. Furthermore, the enhanced colocalization of MAVS with mitochondria in stimulated UC-MSCs supports the essential role of MAVS-mitochondrial signaling in antiviral responses, as previously observed in fibroblasts and macrophages [45].

Upon MAVS activation, IRF3 and NF-κB play pivotal roles in mediating antiviral responses. Our data indicate significant IRF3 and NF-κB p65 phosphorylation following 3p-hpRNA stimulation, suggesting a conserved RIG-I-mediated signaling mechanism across multiple cell types, including MSCs [21,31,46]. This activation led to increased production of type I and III IFNs and the transcription of ISGs and inflammatory cytokines, reinforcing the immunomodulatory and antiviral properties of UC-MSCs [24]. The upregulation of IFN-I and IFN-III observed in UC-MSCs is consistent with findings in fibroblasts and lung epithelial cells infected with IAV, where these cytokines are essential for mounting an antiviral response [47]. Additionally, ISGs such as ISG15, OAS2, TRIM22, and USP18 were significantly upregulated, highlighting the antiviral capacity of UC-MSCs, as was described for bone marrow-derived MSCs and fibroblasts [48,49]. Our findings align with reports from epithelial and dendritic cells, where RIG-I agonists, such as poly(I:C) or 3p-hpRNA, drive the expression of ISG15, IFI27, and OAS2, enhancing antiviral defenses [50,51].

Interestingly, although canonical NF-κB downstream targets such as IL-6, IL-8, IL-10, and TNF were upregulated following 3p-hpRNA stimulation, IL-1β levels remained unchanged. While this may initially appear unexpected, given that IL1B is a classical NF-κB target gene, it is consistent with the current understanding of the two-signal model required for IL-1β production. In this model, RIG-I/MAVS activation delivers the first signal, initiating pro-IL-1β transcription; however, the lack of a second activating signal—such as inflammasome assembly (e.g., via NLRP3), ATP release, or mitochondrial distress—prevents the processing and secretion of the mature, bioactive cytokine. Supporting this notion, Lin et al. demonstrated that foreskin-derived stromal cells (FDSCs) can activate ISGs and produce IFN in response to viral stimulation or RIG-I agonists without mounting a vigorous inflammatory response [52]. Notably, IL-1β remained undetectable or very low under those conditions. These findings collectively suggest that UC-MSCs, like FDSCs, can elicit a robust antiviral program while avoiding excessive inflammation, a feature that aligns well with their known immunomodulatory and protective roles.

Several studies have employed polyinosinic–polycytidylic acid (Poly(I:C)), a synthetic analog of double-stranded RNA, as a potent agonist of both RLRs and TLR3, to model antiviral responses in MSCs and other cell types [27,52,53,54]. These investigations reported the activation of IRF3- and NF-κB-dependent pathways, leading to the induction of type I IFN and pro-inflammatory mediators. For instance, Yang et al. (2013) demonstrated that the stimulation of mouse bone marrow-derived MSCs with Poly(I:C) resulted in increased expression of IFN-β and IL-6, supporting the involvement of RLR signaling in the regulation of inflammatory and antiviral genes [27]. Similarly, Raicevic et al. (2017) showed that the transcriptional response to Poly(I:C) or 3pRNA is cell-source-dependent, with distinct cytokine and IFN expression profiles observed across MSCs derived from bone marrow, adipose tissue, and umbilical cord [54]. Specifically in UC-MSCs, the authors observed an upregulation of IFN-β, IFN-λ1, and IL-6, along with additional evaluation of TNF-α and IL-1α, suggesting that these cells can mount a balanced pro-inflammatory and antiviral response upon RLR stimulation. Notably, Lin et al. also used Poly(I:C) in foreskin-derived stromal cells and confirmed the activation of RLR/IRF3 pathways [52], further reinforcing the relevance of this agonist as a tool to study innate immune responses in non-immune stromal cells.

A key question in our study was whether MAVS activation affects mitochondrial function in UC-MSCs. We utilized multiple approaches to address this, including live-cell metabolic analysis, mitochondrial stress tests, and oxidative stress assays. Seahorse XF analysis confirmed that the OCR remained stable, suggesting no metabolic shift upon MAVS activation. Additionally, there are no significant changes in cell viability, ATP production, ROS levels, and ΔΨm, further supporting the mitochondrial bioenergetics stability. These comprehensive analyses suggest that UC-MSCs possess an intrinsic resilience to RIG-I/MAVS-mediated antiviral responses, maintaining their mitochondrial function despite robust immune activation.

Previous studies have reported that prolonged MAVS activation, particularly in response to viral infections or RLR stimulation, disrupts mitochondrial dynamics and increases ROS levels in hepatocytes and epithelial cells, ultimately impairing bioenergetics [55]. While other MSC types, such as BM-MSCs and adipose-derived stem cells (ADSCs), undergo metabolic reprogramming and mitochondrial remodeling during differentiation or inflammation [56], UC-MSCs maintained a stable mitochondrial morphology and function following stimulation. We evaluated mitochondrial dynamics to assess this stability further. Stimulation with various viruses, such as IAV and respiratory syncytial virus (RSV), or synthetic RLR agonists like poly(I:C), has been shown to trigger mitochondrial fragmentation, increased fission, and altered bioenergetics in MSCs, epithelial cells, and macrophages, often mediated by dysregulation of DRP1 and OPA1 [57,58,59]. In contrast, our results indicate that UC-MSCs preserve mitochondrial integrity upon MAVS activation. Protein expression analysis revealed no significant alterations in the expression of mitochondrial dynamics regulators such as OPA1 and DRP1, as also supported by TEM observations that showed preserved mitochondrial morphology. Our findings indicate that the resistance to mitochondrial dysfunction upon MAVS activation by 3p-hpRNA may represent a distinctive biological property of UC-MSCs, potentially associated with their tissue origin or developmental stage. However, further comparisons with other cell types are needed to determine whether this resilience is unique to MSCs or a shared characteristic among specific cell populations [60,61].

It is generally accepted that the reparative function of MSCs is partly mediated by mitochondrial transfer, which seems to be an evolutionarily conserved phenomenon [30,62]. Mitochondrial transfer from MSCs to host cells has been identified as a mechanism for restoring cellular bioenergetics, cell survival, and tissue repair [30,62,63]. Our study demonstrates that UC-MSCs maintain their mitochondrial transfer capacity after 3p-hpRNA stimulation, as evidenced by the co-culture with lung epithelial cells. This function preservation suggests a therapeutic advantage, as many virus-infected cells experience mitochondrial dysfunction as a viral evasion strategy [64,65].

Additionally, we evaluated the function of isolated mitochondria from UC-MSCs post-stimulation with 3p-hpRNA. Morphological analyses by TEM revealed no significant differences, and ATP production and ΔΨm values remained stable, further supporting the integrity of mitochondrial bioenergetics. Mitochondrial transplantation, which uses isolated functional mitochondria, is being studied in preclinical and clinical trials as a potential treatment for various diseases related to mitochondrial dysfunction, including sepsis, ischemia, obesity, diabetes, and liver disease [66,67,68,69]. Our study showed that isolated UC-MSC-derived mitochondria from both control and 3p-hpRNA-stimulated cells retained their ability to be transferred to recipient cells such as A549. Transplanting these mitochondria into epithelial cells resulted in a cell-conserved viability, reduced oxidative stress, and enhanced ΔΨm in all the mitochondrial conditions (MT, MT^2h^, and MT^24h^). This suggests that transferring isolated or exogenous mitochondria from UC-MSCs exposed to certain viral conditions or after MAVS activation will be possible with functional mitochondria, reinforcing their potential application in a viral and inflammatory context. Further studies are needed to determine whether similar effects are observed in other cell types and in vivo viral infection models.

## 4. Materials and Methods

### 4.1. Isolation and Culture of Umbilical Cord-Derived Mesenchymal Stromal Cells (UC-MSCs)

UC-MSCs were isolated, cultured, expanded, and fully characterized according to international guidelines [70]. Briefly, UC-MSCs were cultured in Dulbecco’s Modified Eagle’s Medium-low glucose (DMEM-LG), supplemented with 1% penicillin/streptomycin solution (10,000 U/mL/10,000 mg/mL), 1% L-glutamine (200 mM), and 10% fetal bovine serum (FBS, all from Gibco, Waltham, MA, USA). The cells (passages P5-P6) were incubated at 37 °C with 5% CO_2_ until 80–90% confluency. All UC-MSCs tested negative for Mycoplasma (PCR Mycoplasma Detection Kit, abm, Richmond, BC, Canada).

For co-culture experiments, UC-MSCs and A549 epithelial lung cells (CCL-185, ATCC, Manassas, VA, USA) were labeled with MitoTracker Green FM and MitoTracker Red CMXRos, respectively (Thermo Fisher Scientific, Waltham, MA, USA). Briefly, cells were incubated with 200 nM of the respective MitoTracker dye in growth medium for 20–30 min at 37 °C, washed twice with PBS, and gently detached using TrypLE Express (Thermo Fisher Scientific) (5 min, 37 °C). Labeled cell pellets were resuspended in fresh complete DMEM, mixed at a 1:1 ratio, and co-cultured for 24 h at 37 °C in a 5% CO_2_ incubator. Monocultures of each cell type—labeled with the corresponding dye but without the co-culture partner—were maintained in parallel as controls for flow cytometry compensation and fluorescence channel gating.

### 4.2. Transfection and Stimuli with 3p-hpRNA

To achieve stimulation of the RIG-I pathway, UC-MSCs were treated with 3p-hpRNA (Invivogen, San Diego, CA, USA), which was delivered into the cell cytoplasm, using the transfection agent, LyoVec™ (Invivogen, San Diego, CA, USA), following the manufacturer’s instructions. 3p-hpRNA is a 5′ triphosphate hairpin RNA generated from the influenza A (H1N1) virus, a single-stranded negative-sense RNA virus. Once UC-MSCs reached 80–90% confluence, they were transfected directly with the LyoVec+3p-hpRNA complex (250 ng/mL) for 2 and 24 h. 3p-hpRNA was chosen as the RIG-I agonist due to its high specificity and well-characterized role in triggering MAVS-dependent antiviral responses. Unlike poly(I:C), which can activate multiple pattern recognition receptors (PRRs) such as TLR3 and MDA5, 3p-hpRNA selectively engages RIG-I, making it an ideal tool for dissecting MAVS-specific signaling pathways in UC-MSCs.

### 4.3. Microscopy

Confocal microscopy of UC-MSCs treated with 3p-hpRNA was carried out using a Carl Zeiss LSM 700 confocal laser system (Thermo Fisher Scientific, Waltham, MA, USA). Mitochondria were labeled with MitoTracker Green (Thermo Fisher Scientific), MAVS were detected with a specific antibody for human MAVS (D5A9E, Rabbit mAb 1:200; Cell Signaling Technology, Danvers, MA, USA) with a secondary antibody (Alexa Fluor™ 555 Rabbit 1:5000, ThermoFisher Scientific), and the cytoplasm was stained with Phalloidin (Alexa Fluor™ 635 Phalloidin, 0.1 µM, ThermoFisher Scientific).

Transmission electron microscope (TEM) images were acquired with a Talos F200C (ThermoFisher Scientific) transmission electron microscope at 200 kV at the Advanced Microscopy Unit at the Pontificia Universidad Católica de Chile. UC-MSCs and isolated mitochondria-enriched fractions were fixed with fresh glutaraldehyde and mounted onto formvar-coated copper grids (300 mesh, Ted Pella Inc., Redding, CA, USA). Samples were negatively stained with 2% phosphotungstic acid (Sigma-Aldrich, Merck KGaA, Darmstadt, Germany).

### 4.4. Mitochondria-Enriched Fraction Isolation

UC-MSC pellets (1 × 10^6^) were obtained, and mitochondrial-enriched fractions were isolated using the Mitochondria Isolation Kit for Mammalian Cells (ThermoFisher Scientific) as described previously [71]. Briefly, the cell pellet was homogenized in kit reagent A, and a protease inhibitor was added, followed by several homogenization and centrifugation steps to obtain the mitochondrial-enriched fraction.

### 4.5. Determination of Inflammatory Mediators

Levels of IFN types I, II, and III, interleukin (IL)-1β, IL-6, IL-8, and IL-10, and TNFα were determined as previously [72] in UC-MSC supernatant stimulated for 2 and 24 h, using commercial ELISA kits (R&D Systems, Minneapolis, MN, USA) according to the manufacturer’s instructions.

### 4.6. Apoptosis Assay

According to previous work [72], cells (1 × 10^5^ cells) were stained with 0.5 μL of Annexin V APC (BioLegend, San Diego, CA, USA) and 7-aminoactinomycin D (7AAD) viability staining solution (BioLegend, San Diego, CA, USA) at 1 μg/mL in 100 μL of Annexin V binding buffer (BioLegend, San Diego, CA, USA) by incubating for 20 min at room temperature. Then, 100 μL of Annexin V binding buffer was added, and the cell suspension was analyzed on a BD FACS Canto II (Becton Dickinson, San Diego, CA, USA) flow cytometer. Data were analyzed using the FlowJo™ Software version 10.

### 4.7. Total and Mitochondrial Oxidative Stress Determination

Total reactive oxygen species (ROS) were detected using the DCF method, employing dichlorofluorescin diacetate (H2DCFDA, ThermoFisher Scientific). Briefly, 1 × 10^4^ cells were seeded in a 12-well plate. The medium was removed, and H2DCFDA (10 µM) was added and incubated for 2 h at 37 °C. Then, the H2DCFDA was removed, the wells were washed, and, in a mirror well as a positive control, H2O2 (150 µM) was added and incubated for 20 min at 37 °C. The wells were washed, and the cells were obtained. To determine live/dead cells, a Fixable Near-IR Dead Cell Stain Kit (1:800) (ThermoFisher Scientific) was incubated for 10 min at room temperature.

Superoxide was detected using the MitoSOX method (Invitrogen, Waltham, MA, USA) according to the manufacturer’s instructions. Briefly, 5 × 10^3^ cells were seeded in a 96-well plate. The medium was discarded, and MitoSOX (1 µM) was added and incubated for 30 min at 37 °C. Then, the MitoSOX was removed, the wells were washed, and, in a mirror well as a positive control, 1 µM Rotenone (Rot, Sigma-Aldrich) was added and incubated for 20 min at 37 °C. The wells were washed, and the cells were obtained. To determine live/dead cells, LIVE/DEAD™ Fixable Yellow (1:800 dilution in 1X PBS; Thermo Fisher Scientific, Waltham, MA, USA) was applied for 10 min. Cells were washed in 1X PBS, and the pellets were collected and resuspended in 1X PBS for analysis. The cell suspension was analyzed on a BD FACS Canto II (Becton Dickinson, Franklin Lakes, NJ, USA) flow cytometer. Data were analyzed using the FlowJo™ Software, version 10.

### 4.8. Mitochondrial Membrane Potential (ΔΨm) Determination

Mitochondrial membrane potential was analyzed in cells treated with 3p-hpRNA and controls using tetramethylrhodamine (TMRE, 400 nM, ThermoFisher Scientific). This cell-permeant dye accumulates in active mitochondria with intact membrane potentials. UC-MSCs were collected and washed with 1X PBS. Then, the cells were stained with TMRE (400 nM) for 30 min at 37 °C. Following staining, the cells were pelleted, washed with 1X PBS, and incubated with LIVE/DEAD™ Fixable Yellow (1:800 dilution in 1X PBS; ThermoFisher Scientific) for 10 min. Cells were washed in 1X PBS, and the pellets were collected and resuspended in 1X PBS for analysis. Carbonyl cyanide 3-chlorophenylhydrazone (CCCP, 10 ng/mL, Sigma-Aldrich), a mitochondrial depolarizing agent, was used as a positive control. Analysis was performed by flow cytometry using a BD FACS Canto II (Becton Dickinson) flow cytometer. Data were analyzed using the FlowJo™ Software, version 10.

### 4.9. ATP Production Analysis

ATP analysis was performed using the CellTiter Glo^®^ Luminescent Cell Viability Kit (Promega, Madison, WI, USA), following the manufacturer’s instructions [73]. A total of 5000 UC-MSCs treated with 3p-hpRNA and controls were seeded into each well of a 96-well white microplate for 12 h. The medium was removed in mirror wells, and the new medium was added with 10 µM Oligomycin A (OLN, Tocris Bioscience, Bristol, UK) for 20 min. The plate was incubated at room temperature for 30 min to equilibrate. Subsequently, 100 μL of the reagent from the kit was introduced into each well, and the plate was placed on an orbital shaker at 140 rpm for 2 min. Then, the ATP analysis was performed by measuring luminescence in a Biotek^®^ FLx800 luminometer (Agilent Technologies, Santa Clara, CA, USA). Results were normalized per µg of total protein of each well.

### 4.10. Seahorse XF HS Mini Analyzer

A multiparameter metabolic analysis of cells treated with 3p-hpRNA and controls was performed in an extracellular flux analyzer (Agilent Technologies, Santa Clara, CA, USA) as previously described [73]. Before 2 and 24 h of 3p-hpRNA-transfection, UC-MSCs were seeded on XF HS Mini 8-well plates and kept overnight at 37 °C in 5% CO_2_ with DMEM-LG. After 24 h, the cell culture medium was replaced with Agilent Seahorse XF DMEM medium pH 7.4 (with 5 mM HEPES) with 10 mM glucose 1 h before the assay. Mitochondrial real-time measurements of the oxygen consumption rate (OCR) were evaluated using 2 µM OLN (Tocris Bioscience), 200 nM FCCP (Sigma-Aldrich), 1 µM rotenone (Rot, Sigma-Aldrich), and 1 µM antimycin-A (AA, Sigma-Aldrich, Merck KGaA, Darmstadt, Germany).

### 4.11. RNA Extraction and RT-qPCR

Total RNA was extracted using TRIzol (ThermoFisher Scientific) from cultured MSCs according to the manufacturer’s instructions. RNA (500 ng), as described previously [5], was reverse transcribed by using a superscript II kit (Invitrogen, Waltham, MA, USA), and qPCR was performed at Stratagene Mx3000P (Agilent Technologies, Santa Clara, CA, USA) with specific primers (Table 1) or Taqman probes (ThermoFisher Scientific) (Table 2). All values were normalized to β-actin as a housekeeping gene and expressed as fold change or relative expression using the 2^−ΔΔCT^ formula [74].

### 4.12. Western Blot Analyses

MAVS, NF-κB1 p105/p50, NF-κB p65, phospho-NF-κB p65, IRF3, phospho-IRF3, Mitofusin 1, Mitofusin 2, OPA 1, DRP 1, phospho-DRP1, and Parkin protein expression levels were determined by Western blotting as a standard protocol [72]. Briefly, cell pellets were homogenized in 1X RIPA lysis buffer (Thermo Fisher Scientific) with a 1% *v*/*v* protease and phosphatase inhibitor cocktail (Thermo Fisher Scientific). Total protein concentrations were determined with a Pierce BCA Protein Assay Kit (Thermo Fisher Scientific), and 20 uL of each lysate was mixed with Laemmli buffer 5X, heated for 5 min at 95 °C, separated on 10% polyacrylamide gels by SDS-PAGE, and transferred to PVDF membranes (GE Healthcare Limited, Chicago, IL, USA).

After one hour of an appropriate blocking step (Intercept Blocking Buffer, LI-COR Biosciences, Lincoln, NE, USA), the membranes were incubated with primary antibodies in a 0.1% Tween 20 solution in 1X TBS overnight, with constant agitation at 4 °C. Primary antibodies used, all from Cell Signaling Technology, were MAVS (D5A9E, Rabbit mAb 1:1000), NF-κB1 p105/p50 (D4P4D, Rabbit mAb, 1:1000), NF-κB p65 (L8F6, Mouse mAb, 1:1000), pNF-κB p65 (93H1, Rabbit mAb, 1:1000); IRF3 (D83B9, Rabbit mAb, 1:1000), Mitofusin 1 (D6E2S, Rabbit mAb 1:1000), Mitofusin 2 (D2D10, Rabbit mAb 1:1000), OPA 1 (D7C1A Rabbit mAb, 1:1000), DRP 1 (4E11B11 Mouse mAb, 1:1000), pDRP 1 (Ser616, D9A1, Rabbit mAb, 1:200), and Parkin (Prk8 Mouse mAb, 1:1000). Also, we used pIRF3 (Ser386, Rabbit mAb, 1:500; Invitrogen, Waltham, MA, USA) and β-actin (sc-47778, 1:5000; Santa Cruz, Dallas, TX, USA) as normalizers. For fluorescence detection of proteins, a secondary anti-mouse (Alexa Fluor 680, Thermo Fisher Scientific) or anti-rabbit (Alexa Fluor 800, Thermo Fisher Scientific) antibody at a dilution of 1:25,000 in 0.1% Tween 20 in 1X TBS was used. Protein signals were captured using a LI-COR Odyssey imaging system (LI-COR Biosciences, Lincoln, NE, USA) and quantified by densitometric analysis with the ImageJ software, version 1.54b (NIH, Bethesda, MD, USA).

### 4.13. Statistical Analysis

Data were expressed as the mean ± standard error of the mean (±SEM). For the analysis of two or more groups, one-way ANOVA was used with a post hoc test for multiple comparisons, controlling the false discovery rate using the Bonferroni and Hochberg method. All results were analyzed and plotted using GraphPad Prism version 10.1.1 (GraphPad Software Inc., San Diego, CA, USA). For all comparisons, differences were considered statistically significant when *p* ≤ 0.05.

## 5. Conclusions

Our findings demonstrate that MAVS activation in UC-MSCs through 3p-hpRNA induces a robust antiviral response characterized by IRF3 and NF-κB activation, Type I-III IFNs, ISG upregulation, and inflammatory cytokine production, all while maintaining cell mitochondrial function. This balance between immune activation and metabolic integrity underscores the feasibility of UC-MSCs as potential antiviral therapeutic agents. Future studies should further explore MAVS activation in in vivo models to determine its physiological significance. Additionally, given the complexity of antiviral signaling, investigating the roles of other PRRs, such as TLR3 or MDA5, will provide a more comprehensive understanding of UC-MSC-mediated immunity and their clinical potential.

## Figures and Tables

**Table 1:** Primer sequence.

Gene		Sequence 5′-3′
IFN-ß	Fw *	AACTGCAACCTTTCGAAGCC
	Rv *	TGTCGCCTACTACCTGTTGTGC
0AS	Fw	AGAAGGCAGCTCACGAAACC
	Rv	CCACCACCCAAGTTTCCTGTA
β-actin	Fw	CATGTACGTTGCTATCCAGGC
	Rv	CTCCTTAATGTCACGCACGAT
RIG-1	Fw	GCAGGATTTGTAAAGCCCTGTT
	Rv	CACTGATAATGAGGGCATCATTATATTT
IRF3	Fw	GCACAACCTTGACCATCACG
	Rv	ACACATACTGGGCAGTGAGC
MAVS	Fw	GTCACTTCCTGCTGAGA
	Rv	TGCTCTGAATTCTCTCCT
TNF-α	Fw	AGCTGCCCCTCAGCTTGA
	Rv	ATCTTCTCGAACCCCGAGTGA
IL-10	Fw	GTGATGCCCCAAGCTGAGA
	Rv	CACGGCCTTGCTCTTGTTTT
IL-6	Fw	AGTGAGGAACAAGCCAGAGC
	Rv	AGCTGCGCAGAATGAGATGA
UPS	Fw	GCATCGAAGAGTCAAAATAG
	Rv	TTCTTCTCCATTGTCTTCTC
STAT1	Fw	AGCAGAGCTCGTTTAGTGAACC
	Rv	ATTAGGACAAGGCTGGTGGG
IL-1β	Fw	AGGAGCACTTCATCTGTTTAGG
	Rv	CTGAGCTCGCCAGTGAAAT
IL-8	Fw	ACTGAGAGTGATTGAGAGTGGAC
	Rv	AACCCTCTGCACCCAGTTTTC

* Fw: forward; Rv: reverse.

**Table 2:** Taqman probe codes.

Gene	Code
IFNL1	Hs00601677_g1
IFNL3	Hs04193048_gH
IFNA1	Hs03044218_g1
IFI27	Hs01086373_g1
ISG15	Hs01921425_s1
TRIM22	Hs01001179_m1
IFIT1	Hs03027069_s1

## Data Availability

The datasets generated during the present study are available from the corresponding author upon reasonable request.
